# A different method of fault feature extraction under noise disturbance and degradation trend estimation with system resilience for rolling bearings

**DOI:** 10.1371/journal.pone.0287544

**Published:** 2023-07-06

**Authors:** Baoshan Zhang, Jilian Guo, Feng Zhou, Xuan Wang, Shengjun Wei

**Affiliations:** 1 Air Force Engineering University, Xi’an, China; 2 Baoji Titanium Industry Co., Ltd, Baoji, China; Federal University of Pernambuco: Universidade Federal de Pernambuco, BRAZIL

## Abstract

Due to the effects of noise disturbances and system resilience, the current methods for rolling bearing fault feature extraction and degradation trend estimation can hardly achieve more satisfactory results. To address the above issues, we propose a different method for fault feature extraction and degradation trend estimation. Firstly, we preset the Bayesian inference criterion to evaluate the complexity of the denoised vibration signal. When its complexity reaches a minimum, the noise disturbances are exactly removed. Secondly, we define the system resilience obtained by the Bayesian network as the intrinsic index of the system, which is used to correct the equipment degradation trend obtained by the multivariate status estimation technique. Finally, the effectiveness of the proposed method is verified by the completeness of the extracted fault features and the accuracy of the degradation trend estimation over the whole life cycle of the bearing degradation data.

## 1. Introduction

Roll bearings are used in various types of rotary machinery, such as wind turbines and oil drilling equipment. As the degree of degradation increases, the rolling bearings produce a large amount of abnormal vibration accompanied by noise. Moreover, its degradation trend is rather complicated due to the effects of system resilience. Therefore, the research of methods for fault feature extraction (FFE) under noise disturbances and degradation trend estimation (DTE) with system resilience for rolling bearings has always been a hotspot in the field of condition-based maintenance (CBM) [1, 2].

Currently, there are numerous kinds of research on the FFE of rolling bearings under noise disturbances [3, 4], which can be broadly divided into two categories: model-based methods and filtering-based methods. The model-based methods regard faults as abnormal changes that deviate from the normal status [5]. In terms of vibration signals, FFE aims to extract the shock component, which means that the optimal estimation of the degradation trend is obtained by finding the best shock component or kernel function, such as vector autoregressive model [6], local characteristic-scale decomposition [7], Bayesian approach [8, 9]. However, since it is necessary to have a large amount of a priori data or to have an exact equation of status, the fault features under noise disturbances have the characteristics of non-stationarity, nonlinearity, and difficulty to obtain a priori knowledge [10]. As a result, the model-based methods suffer from excessive redundancy in the extracted fault features. The filtering-based methods assume that the low-frequency signal in the vibrational signal is the fault signal, while the high-frequency signal is the noise disturbance that needs to be eliminated. In terms of vibration signals, FFE means finding an optimal filter to separate the noise disturbances from the original signal [11, 12].

The filter-based methods have the advantage of low computational effort and elevated denoising efficiency, which prevents the extracted fault features from being overly redundant, such as wiener filtering [13], wavelet threshold denoising (WTD) [14], and Median filtering [15]. Unfortunately, the filtering-based methods lack a theoretical foundation for their parameter settings, which leads to poor results in FFE. In particular, weak fault features are easily mistaken for noise disturbances in strong noise disturbances.

Among the FFE methods mentioned above, the most widely used is WTD, which includes both hard and soft threshold denoising methods [14]. However, WTD suffers from three shortcomings. The first is the lack of scientific justification for the choice of the number of wavelet decomposition layers. The second is that the wavelet hard threshold function is discontinuous and may give rise to oscillations after noise reduction. The third is that the wavelet soft threshold method has a bias between the processed wavelet coefficients and the true wavelet coefficients, which increases the error when reconstructing the signal. In conclusion, it is particularly crucial to choose a scientific method for evaluating the number of decomposition layers and a suitable wavelet threshold function.

There are two main categories of DTE methods: traditional DTE methods and resilience-based DTE methods, where traditional DTE methods include both parametric and non-parametric regressions. Parametric regression can reflect trends in degradation data, through polynomial interpolation to obtain a function that can fit the degradation data, such as logistic regression [16]. However, the selection of regressors and the expression of regression models are greatly dependent on the researcher’s experience [17], which severely affects the accuracy and generalization ability of regression analysis. In contrast to parametric regression methods, nonparametric regression methods do not make extremely restrictive assumptions about the distribution of the variables and thus arguably extend the range of applications of parametric regression. However, the nonparametric regression has more explanatory variables and is prone to the ‘dimensional disaster’. In terms of process, the multivariate status estimation technique (MSET) is similar to some nonparametric regression analysis methods, such as Nadaraya-Watson regression analysis [18], autocorrelation kernel regression analysis [19], and K Nearest Neighbor (KNN) regression analysis [20], while not requiring artificially set parameters like regression analysis methods. However, its DTE is less effective when the correlation of multivariate fault features is high.

**Fig 1:**
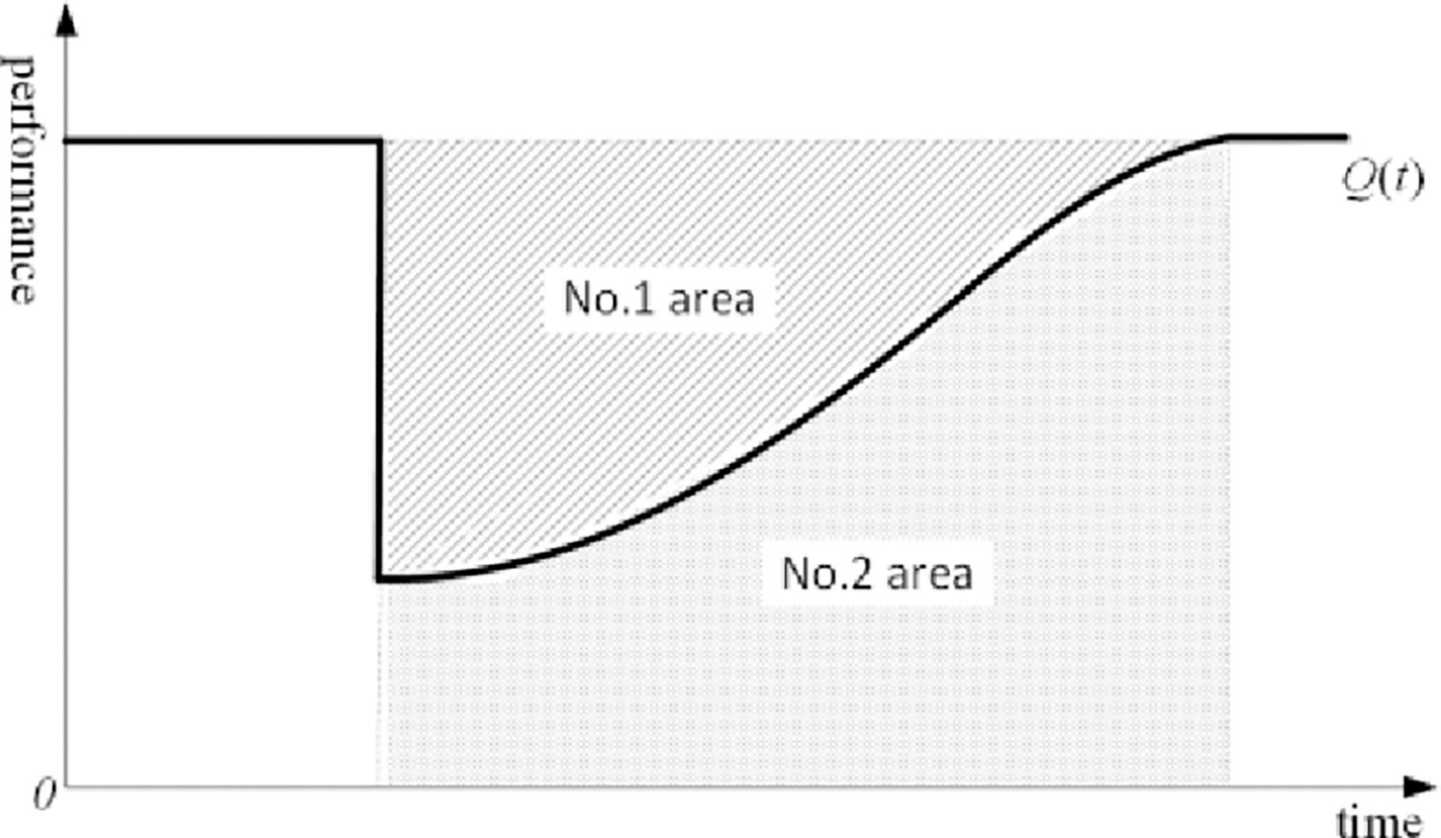
Schematic diagram of the deterministic indexes method.

**Fig 2:**
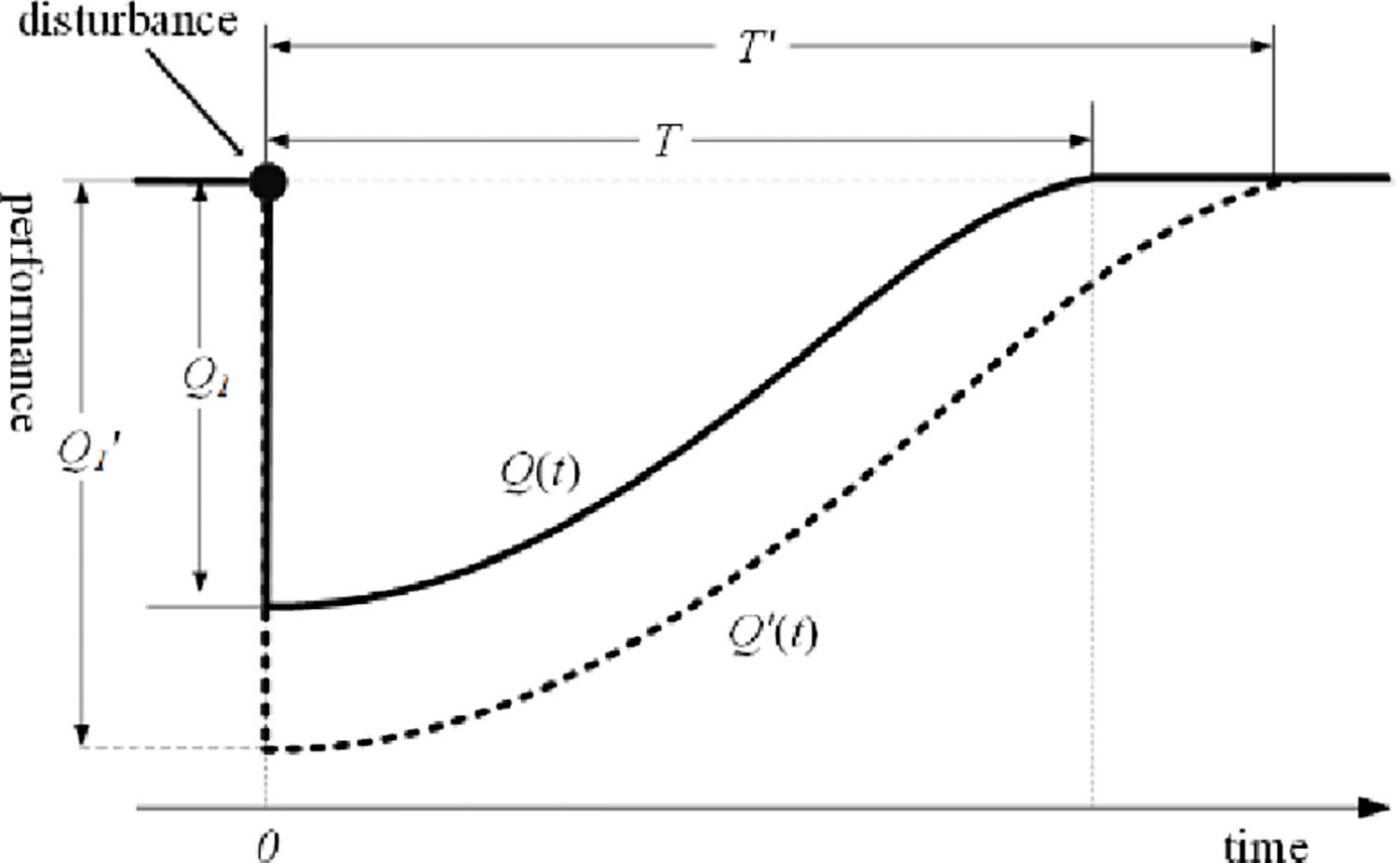
Schematic diagram of probabilistic indexes method.

The resilience-based DTE methods fall into two main categories: deterministic and probabilistic indexes [21]. The key idea of the deterministic indexes method [21–24] is to measure the system resilience based on the cumulative change or loss in system performance after a disturbance. Fig 1 shows a schematic diagram of the deterministic indexes method, where areas 1 and 2 show the loss and residual performance, respectively. In Fig 2, *Q*_1_ and $Q_{1}^{'}$ are the actual maximum performance loss and the given threshold, respectively. *T* and *T*’ are the actual system recovery time and the given threshold, respectively. The probabilistic indexes method [21, 25, 26] regards disturbances, performance loss, and performance recovery as random events, and measures system resilience by considering the randomness of performance degradation and the randomness of recovery time of the system under disturbances.

While the aforementioned methods have achieved great results in system resilience estimation, they are more focused on estimating system resilience at a single time instant, while less research has been done on estimating system resilience at successive times. It is well known that the latter case is closer to reality.

Although the above methods have achieved great results, they all divide the ability of system resilience to cope with change, which is divided into absorption, adaptation and recovery, according to the chronological order [27–31]. They focus on measuring the ability of systems to cope with change in a single moment or instantaneous disturbance [32]. However, it is reality that the disturbances are often continuous. System resilience at any moment of the equipment degradation process is a combination of absorption, adaptation, and recovery. It is clear that the method that divides system resilience by chronological order is no longer fully suitable. Obviously, the influence of system resilience on the degradation trend under continuous disturbance is more focused on the "restorability" during the degradation process.

**Fig 3:**
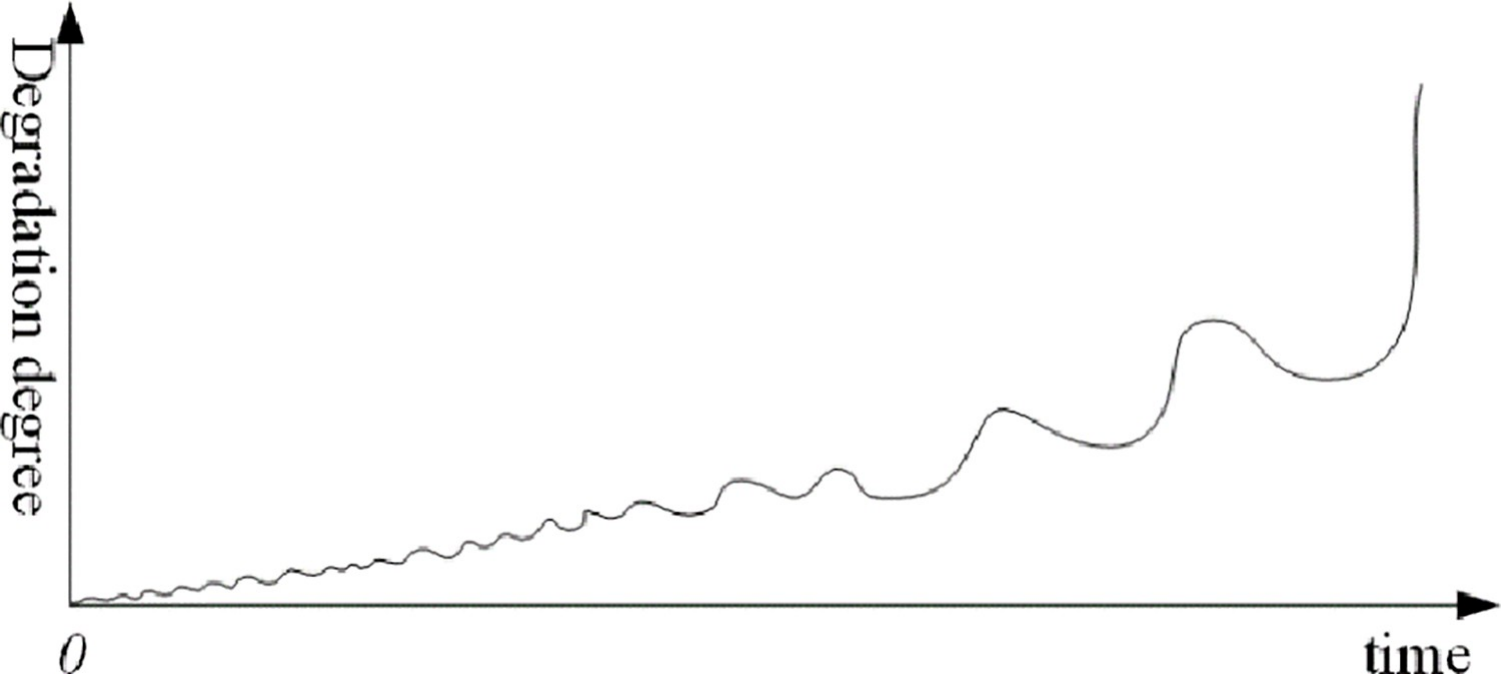
Schematic diagram of the degradation degree of ‘waves’.

Because of the influence of system resilience, the degradation trend is often not an ideal monotonic curve, but a "wave" degradation curve, as shown in Fig 3. Obviously, compared with the research of system resilience under the influence of single moment or instantaneous disturbance, the resilience-based DTE methods under continuous disturbance is obviously closer to reality.

**Fig 4:**
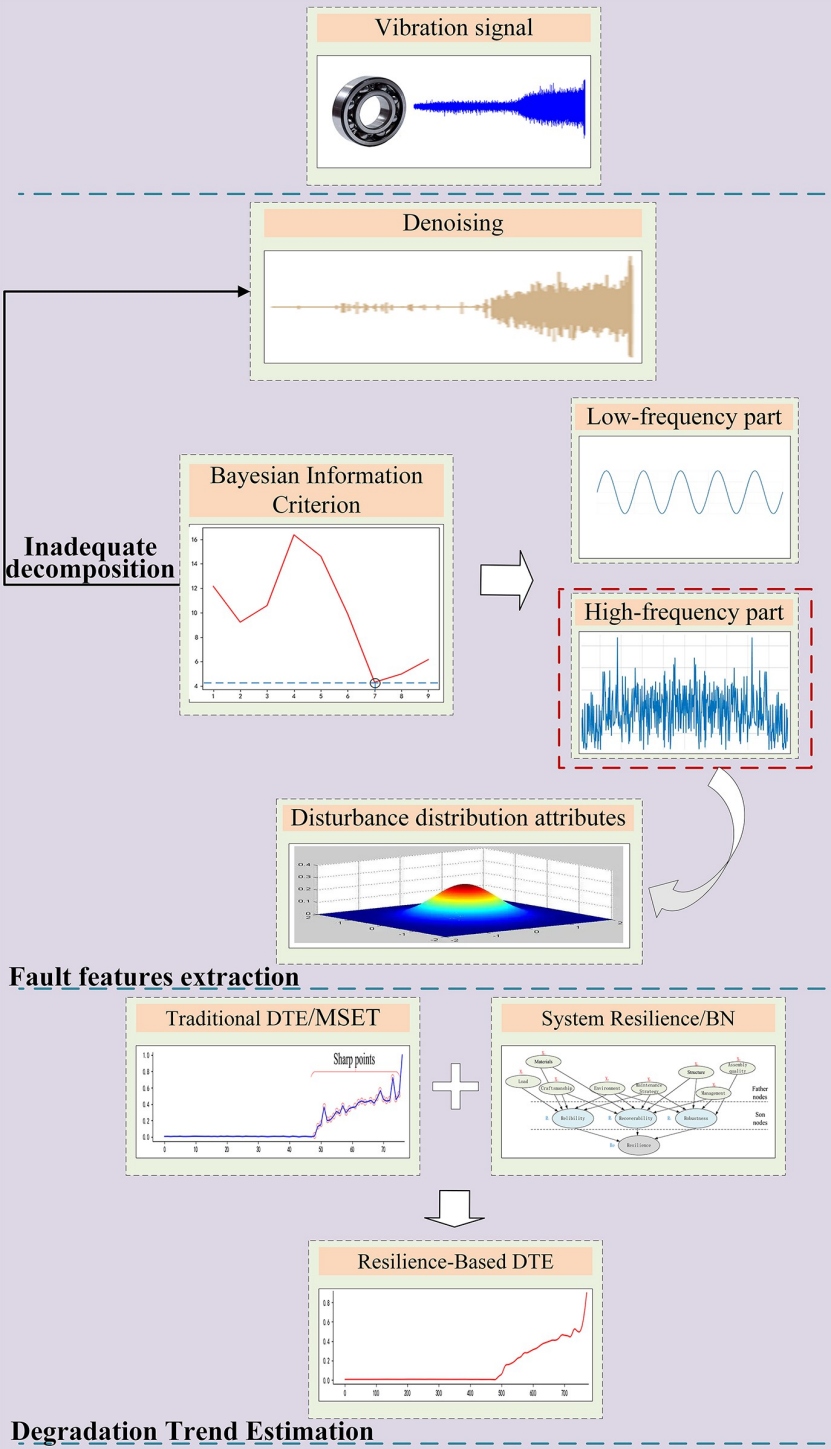
Modeling flowchart.

Based on the above analysis, we propose a different method of FFE under noise disturbance and DTE with system resilience for rolling bearings. The remaining sections of this paper are organized as follows, as shown in Fig 4. Section 2 uses the Bayesian information criterion to judge whether the BIC value of the high-frequency part of the vibration signals is minimized, which means that whether the distribution of the high-frequency part of the data is destroyed, in order to achieve denoising and fault feature extraction. Section 3 firstly, the multidimensional data processing capability of MSET (one of the traditional degradation trend estimation methods) is optimized with the Marxian distance to achieve the degradation trend estimation. Second, we refer to the modeling ideas of stress-strength/damage-endurance reliability models to estimate system resilience with Bayesian networks. Finally, the system resilience is used to correct the MSET. Section 4 verifies the effectiveness of the proposed model with experimental data on the degradation of rolling bearing whole-life performance. Section 5 concludes the paper and plans for subsequent research.

## 2. Fault feature extraction

Since the WTD method has numerous shortcomings, the main work of FFE in this paper focuses on the following two points for achieving a better FFE effect. First, we propose an improved wavelet threshold function to overcome the shortcomings of the traditional WTD method in extracting fault features. Second, the Bayesian information criterion (BIC) is used to evaluate the complexity of the separated high-frequency part of the vibrational signal to decide whether the number of wavelet decomposition layers is appropriate.

The method of FFE based on the improved WTD method is as follows.


ω^j,k={0,|ωj,k|<λsgn(ωj,k)(|ωj,k|−λ+ωj,k2−λ21+e2ωj,kλ),|ωj,k|≥λ (1)

Where *λ* is the threshold, *ω*_*j*,*k*_ is the degradation data wavelet coefficient, ${\hat{\omega }}_{j,k}$ is the predicted wavelet coefficient, *j* is the decomposition scale(satisfying 1≤*j*≤*J*, *J* is the maximum scale), and *sgn()* is the sign function. Since $\underset{\omega_{j,k}\to \lambda^{-}}{\lim}{\hat{\omega }}_{j,k}=0$ when *ω*_*j*,*k*_→λ^−^ and limωj,k→λ+(|ωj,k|−λ+ωj,k2−λ21+e2ωj,kλ)=0 when *ω*_*j*,*k*_→λ^+^, ${\hat{\omega }}_{j,k}$ is continuous in the wavelet domain.

The threshold value *λ* [16] is

$$\lambda =\sigma_{j}\sqrt{2\log(N)}$$ (2)

Where *N* represents the signal length and *σ*_*j*_ represents the standard deviation of noise disturbances in the layer *j*. The expression is

$$\sigma_{j}=\frac{1}{0.6745}*\frac{1}{p}\sum_{i=1}^{n}Cd_{j,k}$$ (3)

Where *Cd*_*j*,*k*_ is the high-frequency part of wavelet decomposition in the layer *j*, *p* is the number of wavelet coefficients at that scale.

The WTD assumes that the degradation data exists in the low-frequency part *Ad*_*j*,*k*_, That is, the noise disturbances exist in the high-frequency part *Cd*_*j*,*k*_. In the ideal case, when the noise disturbances are completely separated from the vibrational signal, the data complexity of the noise disturbances is the lowest. Moreover, the amplitude of the noise disturbances obeys a Gaussian distribution [33]. Therefore, we utilize the BIC to evaluate the complexity of the high-frequency part of the maximum decomposition layer of the vibration signal to judge whether the wavelet threshold function destroys the shape of the amplitude distribution of the noise disturbance, as shown in Eq (4).

BIC=qln(N)−2ln(L) (4)

where *q* is the number of model parameters, *N* is the number of samples, and *L* is the maximum likelihood function obeying Gaussian distribution, that is

$$L(x_{j,k})=\frac{1}{\sigma_{j}\sqrt{2\pi }}\exp[‐\frac{{\left(x_{j,k}-\mu_{j}\right)}^{2}}{2\sigma_{j}{}^{2}}]$$ (5)

If the BIC is minimized, it means that the fault features are separated from the noise disturbances exactly. If the BIC is not minimized, it means that there are not enough decomposition layers for wavelet threshold denoising, which means that the vibrational signal still contains a large amount of noise disturbances, or there are too numerous decomposition layers, which means that some fault features are misclassified as noise disturbances.

## 3. Degradation trend estimation

To compensate for the traditional DTE methods that do not take into account system resilience, the main work on DTE focuses on the following two points. First, the multi-dimensional data processing capability of MSET is optimized by selecting the Mahalanobis distance (MD) that can calculate the similarity between data. Second, the system resilience is constructed to correct for the degradation trend obtained from the improved MSET.

The key idea of MSET [34] is to construct a nonparametric model of the system or equipment, characterize the optimal reconstruction estimate of the observation vector and the historical memory matrix as an estimation vector, and use the discrepancy between the estimation vector and the observation vector to respond to the degradation status of the system or equipment.

Therefore, the FFE at the initial moment is defined as the health status, which is the historical memory matrix in MSET. Moreover, the FFE at subsequent moments is defined as the degradation status, which is the observation vector in MSET. The degradation trend is estimated by calculating the residual value between the observation vector and the historical memory matrix.

Assume there are *n* interrelated variables observed at a certain moment *t*, which will be denoted as the observed variable *X*_*t*_, that is

$$X_{t}={\left[x_{t,1},x_{t,2}\cdots x_{t,n}\right]}^{T}$$ (6)

where *x*_*t*,*n*_ is the observed value of the status variable at moment *t*.

Construct a historical memory matrix *D* with *m* historical moments and *n* associated status variables, that is

$$D=[X_{1},X_{2}\cdots X_{m}]={\left[\begin{array}{cccc} x_{1,1} & x_{1,2} & \cdots & x_{1,m}\\ x_{2,1} & x_{2,2} & \cdots & x_{2,m}\\ \vdots & \vdots & & \vdots \\ x_{n,1} & x_{n,2} & \cdots & x_{n,m} \end{array}\right]}_{n\times m}$$ (7)

The estimated vector *X*_*est*_ is obtained from the linear weighting of the *m* observation vectors *X*_*obs*_ in the historical memory matrix *D*, that is

$$X_{est}=DW=w_{1}X_{1}+w_{2}X_{2}\cdots w_{m}X_{m}$$ (8)

where *W* = [*w*_1_,*w*_2_⋯*w*_*m*_]^*T*^ is an *m*-dimensional vector of weights representing the similarity of the input observation vector *X*_*obs*_ to the historical memory matrix *D*, that is

$$W={\left(D^{T}⊗D\right)}^{-1}(D^{T}⊗X_{obs})$$ (9)

where ⊗ is a nonlinear operator to replace the multiply operation in the matrix for avoiding the irreversible phenomenon generated by *D*^*T*^⊗*D* and expanding the adaptation range of Eq (9) [35]. To enhance the multidimensional data processing capability of the MSET method, the MD between *D*^*T*^ and *X*_*obs*_ [16] is used as the nonlinear operator in MSET in this study, that is

$$⊗(X,Y)=\sqrt{{\left(X‐Y\right)}^{T}\sum^{‐1}(X‐Y)}$$ (10)

Where ∑^-1^ is the inverse matrix of the covariance matrix of a multidimensional random variable, it can be intuitively seen that when the two-status matrices are more similar, their MD is smaller, and when the two-status matrices are more dissimilar, their nonlinear operation results are larger.

Bringing Eq (9) into Eq (8), the final expression of the estimated vector of the MSET model is obtained as

$$X_{est}=D{\left(D^{T}⊗D\right)}^{-1}(D^{T}⊗X_{obs})$$ (11)

The residuals *ε*, which reflect the degraded status, can be visually obtained by comparing the differential values between the observed vector *X*_*obs*_ and the estimated vector *X*_*est*_, that is

$$\varepsilon =X_{est}‐X_{obs}$$ (12)

By comparing the scope of application of various types of failure indexes in Table 1, the root means square value (*RMSV*) is chosen to reflect the degradation status in this study [16].

$$RMSV=\sqrt{\frac{1}{N}\sum_{i=1}^{N}{\left|x_{i}\right|}^{2}}$$ (13)

**Table 1:** Failure index table.

Index	Meaning	Index	Meaning
*VARV*	Describe the fluctuation range of vibration signal	*E*	Characterize the impact of the signal failure
*RMSV*	Denotes signal strength	*SE*	The essential information of bearing fault status is extracted by measuring entropy value effectively
*SF*	The ratios of effective value to rectification mean value	*RE*	
*MF*	Characterize wear degree of mechanical equipment	*TE*	

where *VARV* is the variance value, *RMSV* is the root mean square value, *SF* is the shape factor, *MF* is the margin factor, *E* is the energy, *SE* is the Shannon entropy, *RE* is the Renyi entropy, and *TE* is the Tsallis entropy.

The *RMSV* of the residual *ε* between the *n* dimensions *X*_*est*_ and the *n* dimensions *X*_*obs*_ is used to represent the degradation status indexes *DR*.


$$DR=\sqrt{\frac{1}{m}\sum_{i=1}^{m}{\left|\varepsilon_{i}\right|}^{2}}$$ (14)

**Fig 5:**
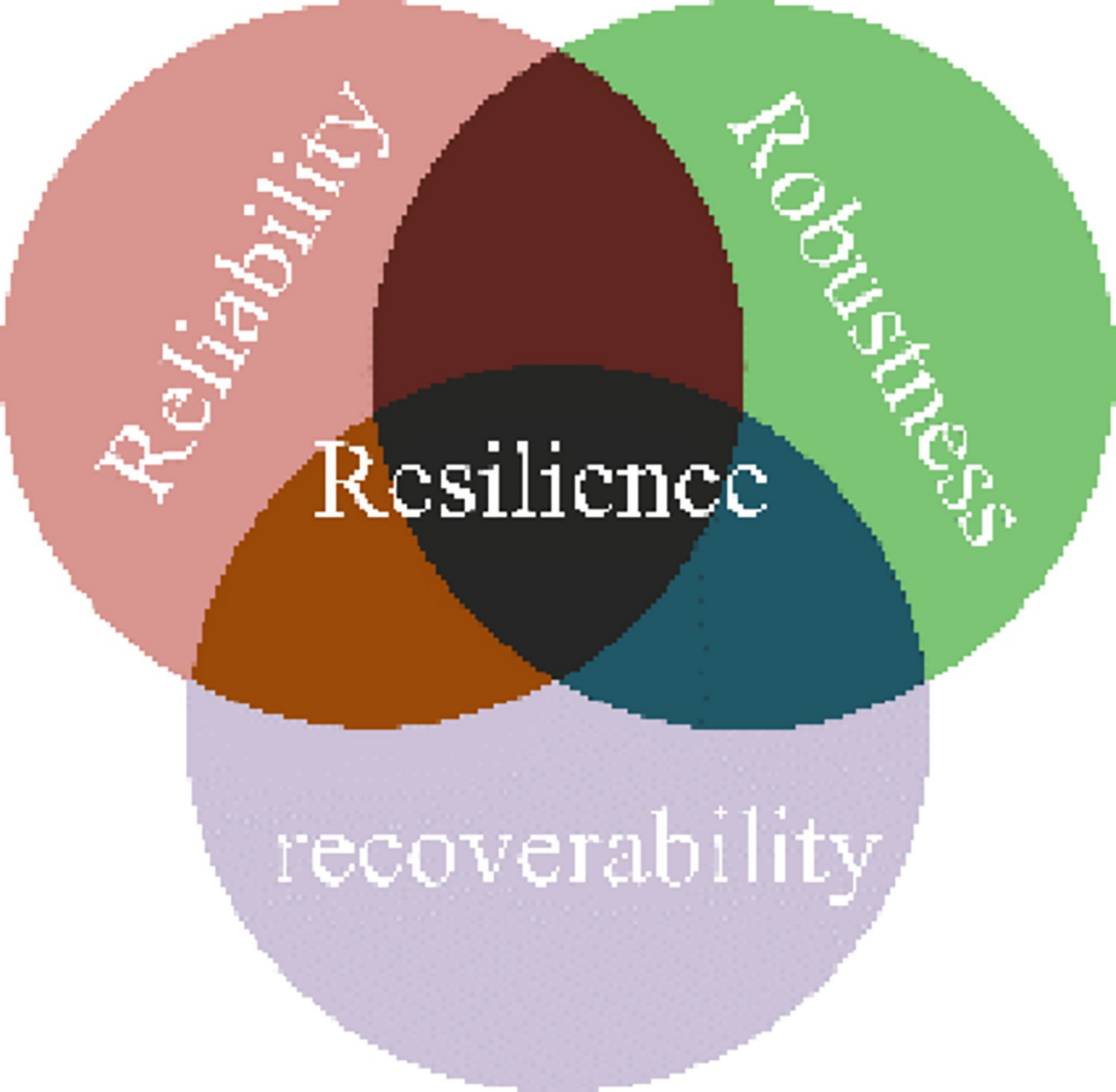
System resilience schematic.

For system resilience, Kharmanda [36] argues that reliability means the capability to avoid failures, Liebchen [37] argues that robustness refers to the capability to maintain performance under adverse conditions, and Piggott [38] argues that recoverability describes the capability to recover from an abnormal status to normal. They respectively represent the capability at one period of the process from potential disturbance to return to normal, while system resilience represents the capability over the whole life cycle. Thus, system resilience is not only the sum of reliability, robustness, and recoverability, but also the result that emerges from the combination of the three [39]. That is, resilience can be described by these three indexes, as shown in Fig 5.

The models of stress-strength and damage-endurance in the Probabilistic physics of failure method to reliability provide a useful reference for the estimation of system resilience [32]. The assumption of the stress-strength model is that if the stress does not exceed its strength, the system or equipment performance does not degrade and failure does not occur. This kind of model has a clear physical meaning and is simple to model. Its assumption is obviously not suitable for degradation processes under continuous disturbance, but more suitable for equipment reliability estimation under a single moment or static load. The assumption of the damage-endurance model is that the stress cumulates the damage metric in an irreversible form. When the cumulative damage metric exceeds a limit, the system or equipment fails. This kind of model takes into account the degradation of system or equipment performance under continuous disturbance. Its generally not based on the failure mechanism, but based on phenomenology or statistics to ensure the reliability of the modeling. Therefore, we estimate the system resilience by Bayesian networks based on the modeling method of stress-strength and the assumptions of the damage-endurance model.

The Bayesian network (BN) [40] is used to analyze and solve the problem of system resilience estimation in this study. It is a probabilistic graphical model that is currently one of the most effective theoretical models in the field of uncertain knowledge representation and inference. The relations of variables in the BN can be expressed as family relations. The nodes represent the random variables, and the directed edges between the nodes represent the mutual relations between the nodes (from the father node to the son node), and the strength of the relations is expressed in terms of conditional probabilities, then the Bayesian probability expression is as follows.

$$P(a_{j}|b)=\frac{P(b|a_{j})P(a_{j})}{P(b)}$$ (15)

where *P*(*a*_*j*_|*b*) is the posterior probability, *P*(*a*_*j*_) is the prior probability [36], *P*(*b*|*a*_*j*_) is the probability that *b* occurs when *a*_*j*_ is true, and *P*(*b*) is the probability that *b* occurs.

**Fig 6:**
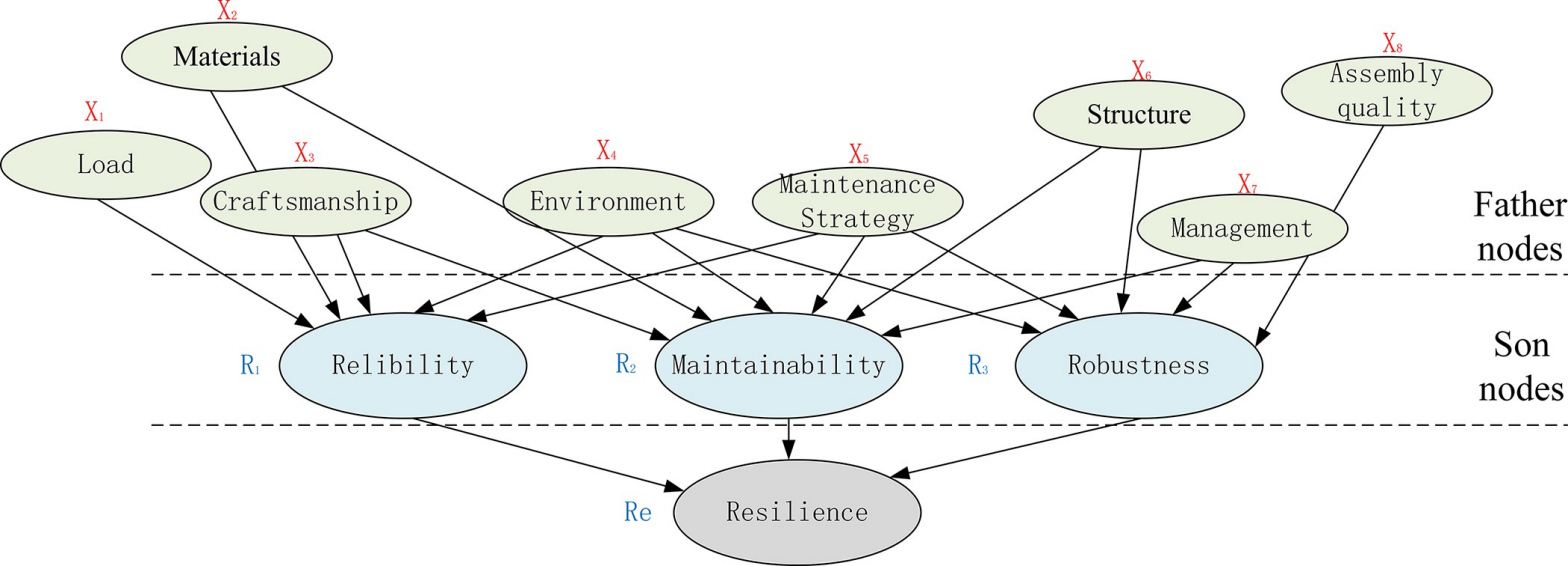
BN to compute system resilience.

The BN method for estimating system resilience is shown in Fig 6.

The father nodes are assigned five levels (High, Good, Medium, Low, and vulnerable) in this study, which respectively corresponds to scores 4, 3, 2, 1, and 0. Son nodes are not assigned a rank, which is computed from the membership function they obey. To obtain the values of the son nodes, a global variable between 0 and 1, as shown in Eq (16), is defined.

x=∑i=1nyi∑i=1nmaxi (16)

where *x* is a global variable, *n* is the number of father nodes corresponding to each son node, *y*_*i*_ is the value of the *i*-th father node, and max_*i*_ is the maximum value of the *i*-th father node.

**Fig 7:**
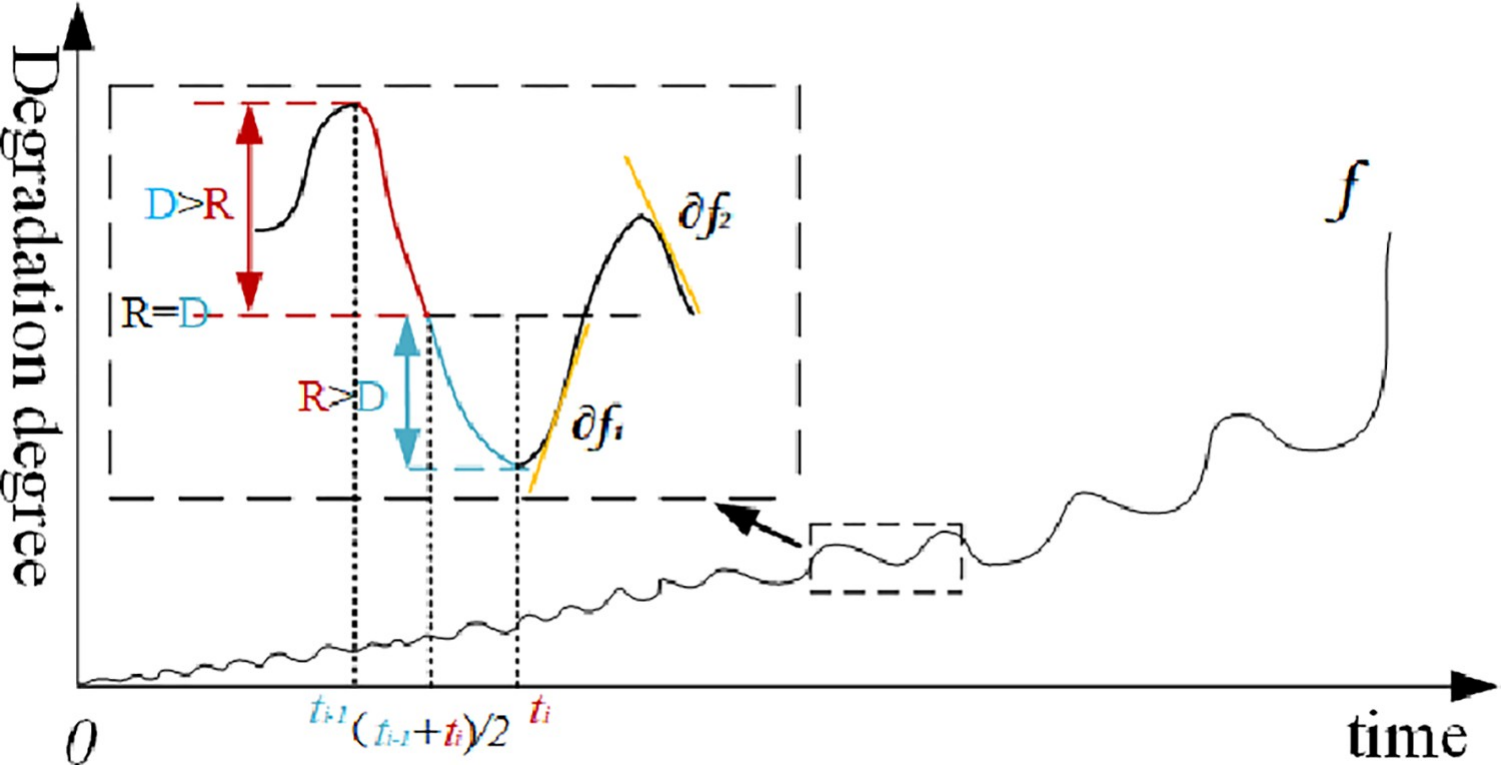
Schematic diagram of the interaction between disturbance and resilience.

Due to the combined effect of the external continuous disturbance and the inner system resilience, the degradation trend obtained by the equipment vibration signal shows the shape of a ‘wave’, that is, a ‘wave’ contains both a process in which the external continuous disturbance *D* is more than the inner system resilience *R* and a process in which the external continuous disturbance *D* is less than the inner system resilience *R*, as shown in Fig 7.

Since the system failure time obeys the generalized gamma distribution in reliability studies [41, 42], the repair time obeys the exponential distribution in recoverability studies [43, 44], and the central limit theorem shows that whatever distribution it originally obeys can eventually be transformed into a normal distribution [45], we use the adjusted generalized gamma distribution, the adjusted exponential distribution, and the normal distribution to represent the membership functions of reliability, recoverability, and robustness of the son nodes respectively.

For the system resilience estimation, which is the joint probability distribution of the BN, by referring to the network relations in Fig 7, the system resilience expression is as follows.

$$\begin{array}{l} SR=P(X_{1})P(X_{2})P(X_{3})P(X_{4})P(X_{5})P(X_{6})P(X_{7})P(X_{8})\\ \;P(R_{1}|X_{1},\cdots ,X_{5})P(R_{2}|X_{2},\cdots ,X_{7})P(R_{3}|X_{4},\cdots ,X_{8})\\ \;P(Re|R_{1},R_{2},R_{3}) \end{array}$$ (17)

where *SR* is the system resilience, *P*(*X*_*i*_) is the probability that the *i*-the father node is true, *P*(*R*_*j*_|*X*_1_,⋯,*X*_*i*_) is the probability that the *j*-th son node is true, and *P*(*Re*|*R*_1_,*R*_2_,*R*_3_) is the probability that the system resilience is true when *R*_1_,*R*_2_,*R*_3_ are all true.

The time for a single sampling of the vibrational signal is short and is dominated by the interplay between external disturbances and internal system resilience. We assume that both the external disturbances and the internal system resilience vary linearly during a single sampling period. This means that the ’peak’ to ’trough’ process contains one phase where the external disturbances are stronger than the internal system resilience and the other phase where the internal system resilience is stronger than the external disturbances, where both phases are centrally symmetric. Moreover, the system resilience decreases as the degree of degradation increases. We define ||∫(ti−1+ti)2tif(t)|−∫(ti−1+ti)2tif((ti−1+ti)2)|SRi as the maximum value that the system can recover at the current degradation degree, by referring to the deterministic index in the study of system resilience. Where *SR* is the proportion of the system that can recover at the current degradation degree, and it will gradually decrease with degradation, which is *SR*_*i*_ = *SR*_*i*−1_(1−*DR*_*i*_). Then, the degradation trend of the system resilience correction is estimated as follows.

DRi'=DRi−αSgn(∂f)||∫(ti−1+ti)2tif(t)|−∫(ti−1+ti)2tif((ti−1+ti)2)|SRi (18)

where *sgn()* is the sign function, ∂*f* is the partial derivative of the degenerate trend *f*, and *α* is an adjustment factor that takes values between 0 and 1.

In this study, Cubic spline interpolation (CSI) [46] is used to estimate the degradation trend, which not only reduces the error between the obtained degradation trend curve and the actual degradation trend curve but also eliminates the ’sharp points’ in the curve where the derivatives do not exist without changing the original degradation trend.

Assume that a total of *n* moments (*t*_0_,*t*_1_,⋯*t*_*n*−1_) of degradation status indexes $\left(DR_{0},DR_{1},\cdots ,DR_{n-1}\right)$, that is, *n* discrete points $\left(t_{0},DR_{0}),(t_{1},DR_{1}),\cdots ,(t_{n-1},DR_{n-1}\right)$, and *n*−1 intervals $\left[(t_{0},t_{1}),(t_{1},t_{2}),\cdots ,(t_{n-2},t_{n-1})\right]$, are obtained by DTE. The key idea of cubic spline interpolation is to construct a cubic equation *f*(*t*) for each small interval, and the cubic spline equation satisfies the following conditions.

1. Every small interval $[t_{i},t_{i+1}],i=0,1\cdots ,n-2$, is a cubic equation *f*(*t*) = *f*_*i*_(*t*)
2. The interpolation condition is satisfied, that is, $f(t_{i})=DR_{i},i=0,1,\cdots ,n-1$
3. The curve is smooth, that is, *f*(*t*), the derivative ∂*f*(*t*), and the second-order derivative ∂^2^*f*(*t*) are all continuous in the interval [*t*_0_,*t*_*n*−1_], that is, the curve is smooth.

Then the cubic spline function *f*_*i*_(*t*) can be constructed as follows.


$$f_{i}(t)=a_{i}+b_{i}(t-t_{i})+c_{i}{\left(t-t_{i}\right)}^{2}+d_{i}{\left(t-t_{i}\right)}^{3},i=0,1,\cdots ,n-1$$ (19)

The *a*_*i*_,*b*_*i*_,*c*_*i*_,*d*_*i*_ are the four unknown coefficients of each interval. To define the cubic spline function for *n*−1 intervals, we need to find 4*n*−4 unknown coefficients: *a*_*i*_,*b*_*i*_,*c*_*i*_,*d*_*i*_. The 4*n*−6 conditions can be obtained based on the continuity of the interpolation as well as the continuity of the differential.


$$\{\begin{array}{c} f_{i}(t_{i})=DR_{i}\\ f_{i}(t_{i+1})=DR_{i+1}\\ \partial f_{i}(t_{i+1})=\partial f_{i+1}(t_{i+1})\\ \partial^{2}f_{i}(t_{i+1})=\partial^{2}f_{i+1}(t_{i+1}) \end{array}$$ (20)

The remaining two conditions are the boundary conditions at *t*_0_ and *t*_*n*−1_.

## 4. Results and discussion

### 4.1 Data analysis

**Fig 8:**
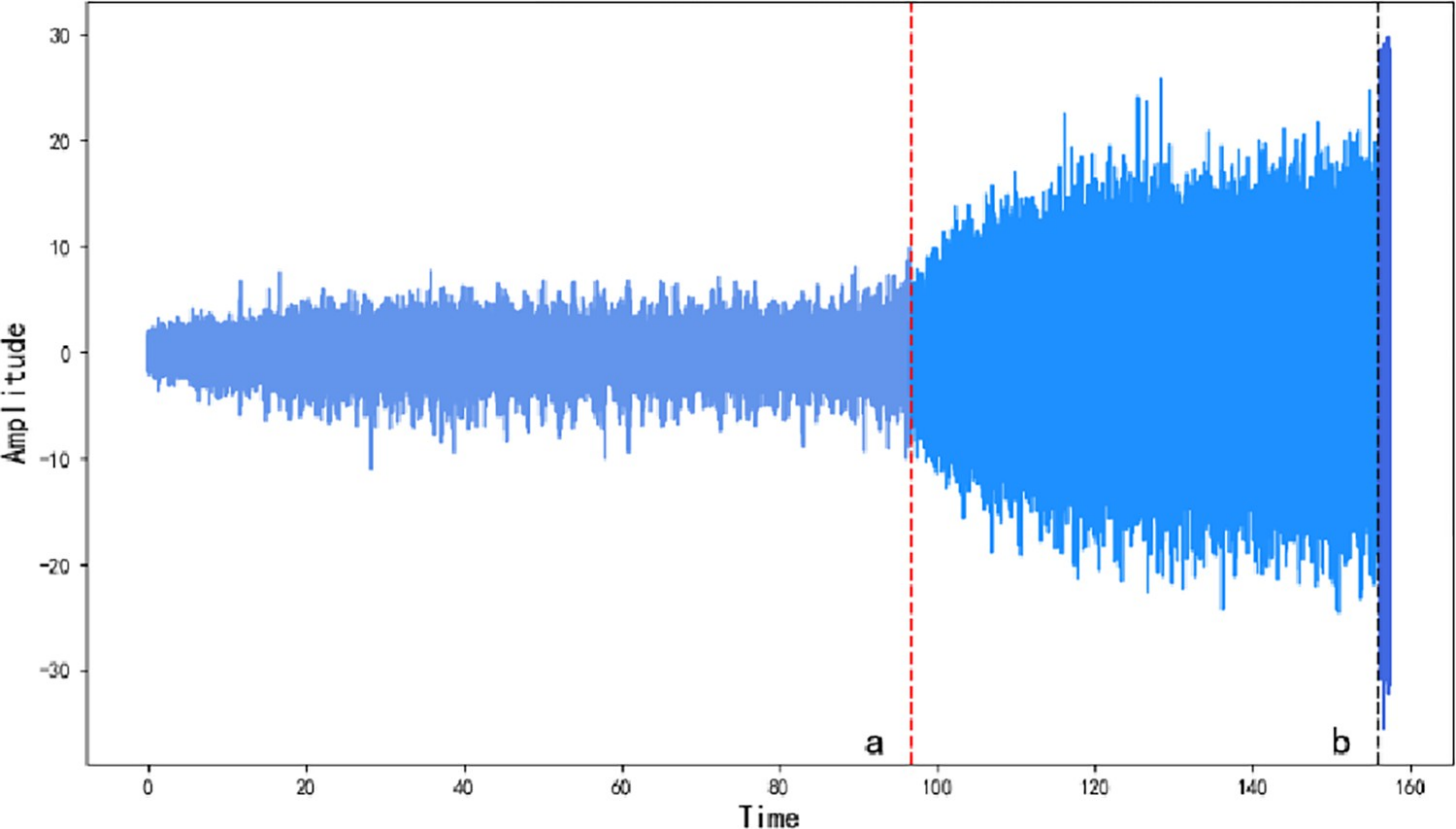
Bearing amplitude time domain chart.

In this paper, we used the bearing degradation data published by Wang Biao et al. [47] from Xi’an Jiaotong University to verify the validity of the proposed method. The sampling frequency was set to 25.6kHz/min, the radial force was 12kN, the rotation speed was 2100rpm, and the operation time was 157.44s. Since the load is applied in the horizontal direction, the accelerometer in this direction can more accurately reflect the degradation information of the tested bearing. Therefore, this study selected the vibration signal in the horizontal direction to reflect the degradation status of the bearing tested. Since the bearing degenerates continuously under a constant external force, the optimal degradation curve should be a monotonically increasing curve. As shown in Fig 8.

As can be seen from Fig 8, the amplitude of the degradation raw data does not change dramatically in the early stage of the bearing degradation experiment, that is, before that point *a*. The bearings are in health status and normal operation stage, while the degradation raw data contains a large number of noise disturbances and little degradation data. The amplitude of the degradation data increases with increasing operation time in the later stage of the degradation experiment, that is, between point *a* and point *c*. The bearing degradation is gradually accelerated, and the degradation raw data contains a large amount of degradation data and noise disturbances data at this time. The amplitude of the degradation raw data increases sharply with increasing operation time in the final stage of the degradation experiment, that is, after this point *c*. Bearing starts to fail and the detected degradation raw data contains a large amount of degradation data and noise disturbances data.

Based on the above analysis, the whole-life data for this bearing is broadly representative. Therefore, the validation of the proposed method in this paper focuses on the following two points.

1. The effectiveness of the proposed FFE method is demonstrated through the following three aspects.
  - Whether the proposed WTD method can significantly reduce the noise disturbances in the vibration signal.
  - When denoising is optimal, the data complexity obtained from BIC is minimized.
  - When the denoising effect is optimal, the amplitude of the noise disturbances is exactly destroyed, and the amplitude of the noise disturbances is minimized at this time. In other words, the noise disturbances are separated from the vibration signal and the fault features are not mistakenly eliminated.
2. Whether the degradation trend can be accurately and quickly assessed under consideration system resilience is the key to verifying the effectiveness of the DTE method proposed in this study.

### 4.2 Fault feature extraction analysis

**Fig 9:**
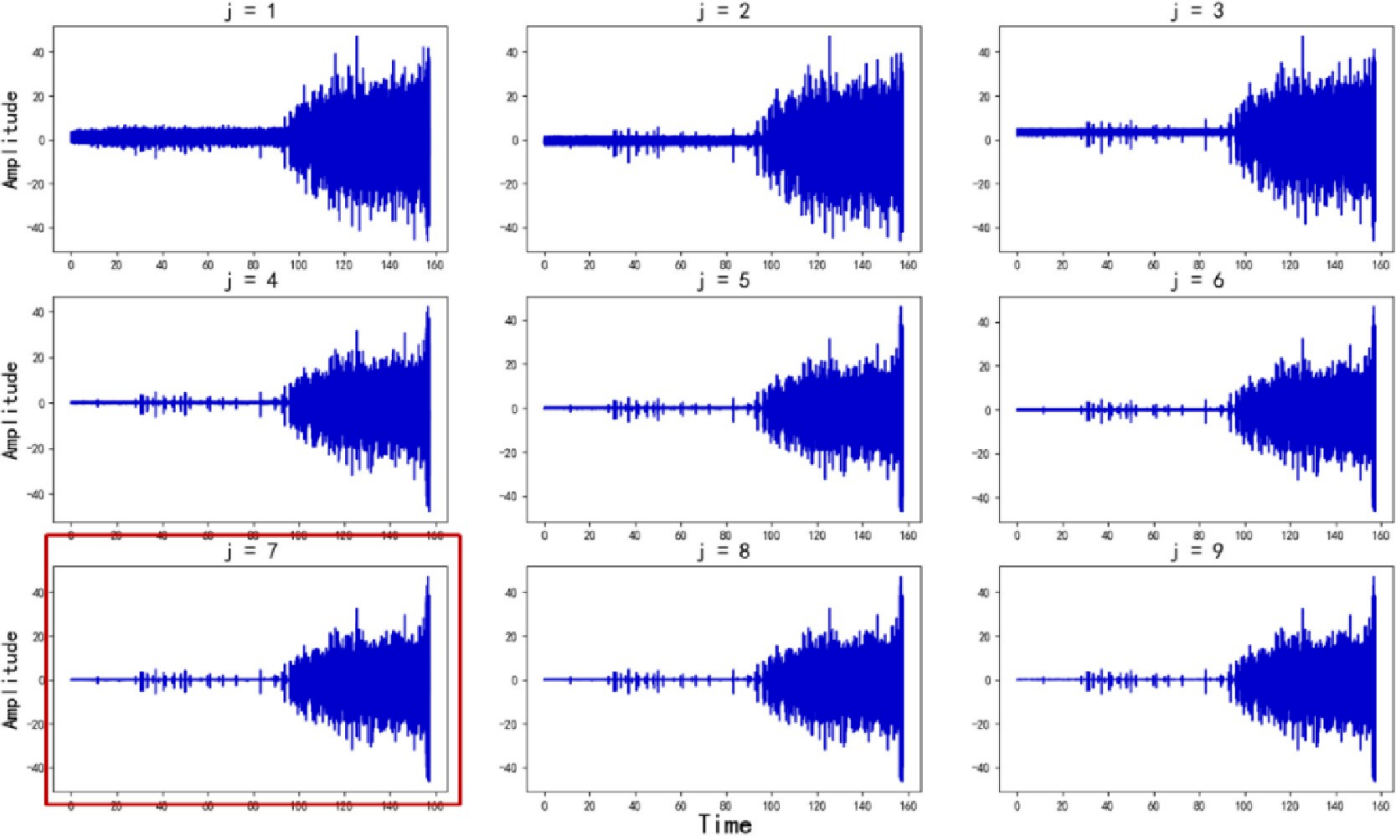
Denoising time domain graph.

**Fig 10:**
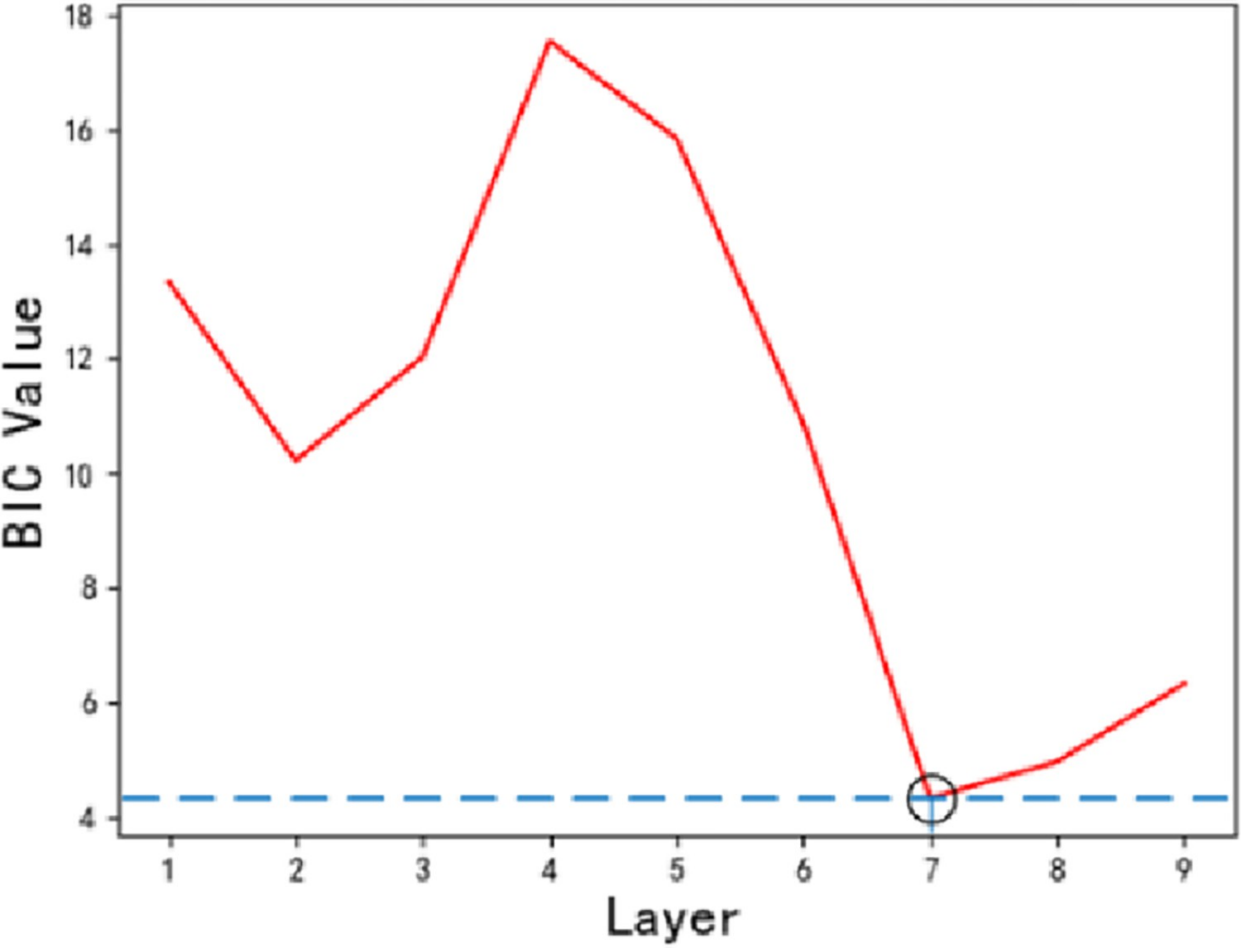
BIC value change graph.

Figs 9 and 10 show the data complexity graph and the denoising effect for each decomposition layer respectively.

We can see that the BIC value is the smallest when the number of wavelet decomposition layers *j* = 7. It can be seen that when the number of wavelet decomposition layers *j*<7, the denoising effect of the improved WTD method is enhanced with the increase of *j*. When the number of wavelet decomposition layers *j*>7, the denoising effect of the improved WTD method is not enhanced with the increase of *j*. Therefore, when *j* = 7, the improved WTD method can not only ensure that the denoising effect meets the requirements but also avoids the elimination of valuable fault features due to the excessive number of decomposition layers. At the same time, we also can see that the BIC introduced in this study can efficiently evaluate the optimal number of decomposition layers for WTD, which demonstrates the effectiveness of the FFE method in this study.

**Fig 11:**
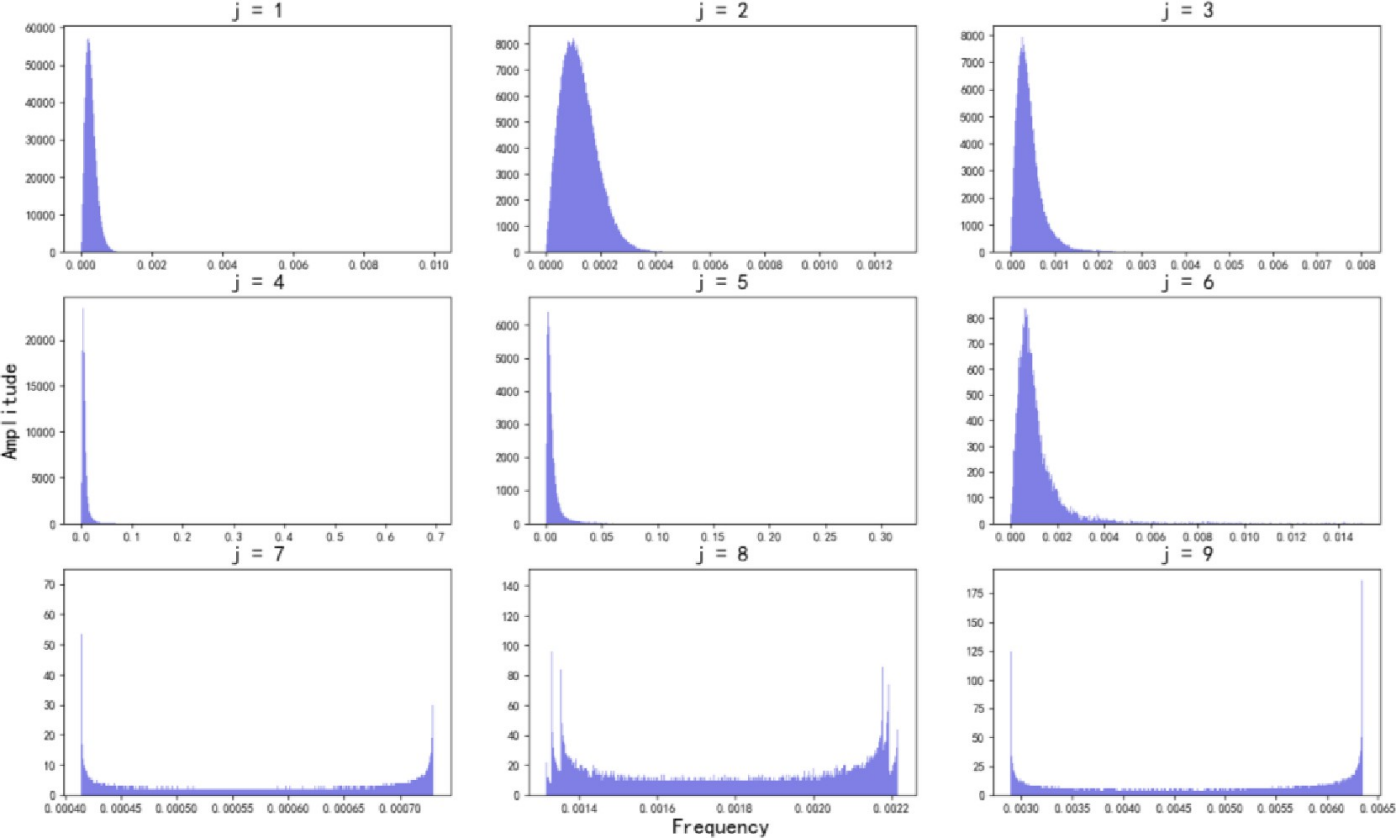
Noise disturbances amplitude distribution graph.

In this study, the amplitude of the noise disturbances obeys the Gaussian distribution as the foundation for assessing the data complexity and FFE method scientificity. The high-frequency part *Cd*_*j*,*k*_ of each decomposition layer is shown in Fig 11.

From Fig 11, it can be seen that when *j*<7, the high-frequency part *Cd*_*j*,*k*_ of the maximum decomposition layers of the improved WTD algorithm obeys Gaussian distribution, which is because the threshold in the improved WTD method has not yet destroyed the distribution characteristics of noise disturbances, meaning that the wavelet decomposition is not sufficient. When *j*≥7, the high-frequency part *Cd*_*j*,*k*_ of the maximum decomposition layers of the improved WTD method does not obey Gaussian distribution, which is because the threshold in the improved WTD method destroys the noise disturbances distribution characteristics. If the decomposition is continued, that is, when the number of decomposition layers exceeds 7, the resulting noise disturbances amplitude increases. It indicates that if the number of decomposition layers continues to increase, the model will mistake some fault features as noise disturbances. In other words, this study evaluates the data complexity by BIC, which can reasonably decide whether the number of FFE is appropriate. Thus, it illustrates the feasibility of setting the BIC in this study to evaluate whether the wavelet decomposition layer is adequate and the rationality of pre-setting the high-frequency part *Cd*_*j*,*k*_ distribution properties of the highest wavelet decomposition layer as the basis for the FFE.

**Fig 12:**
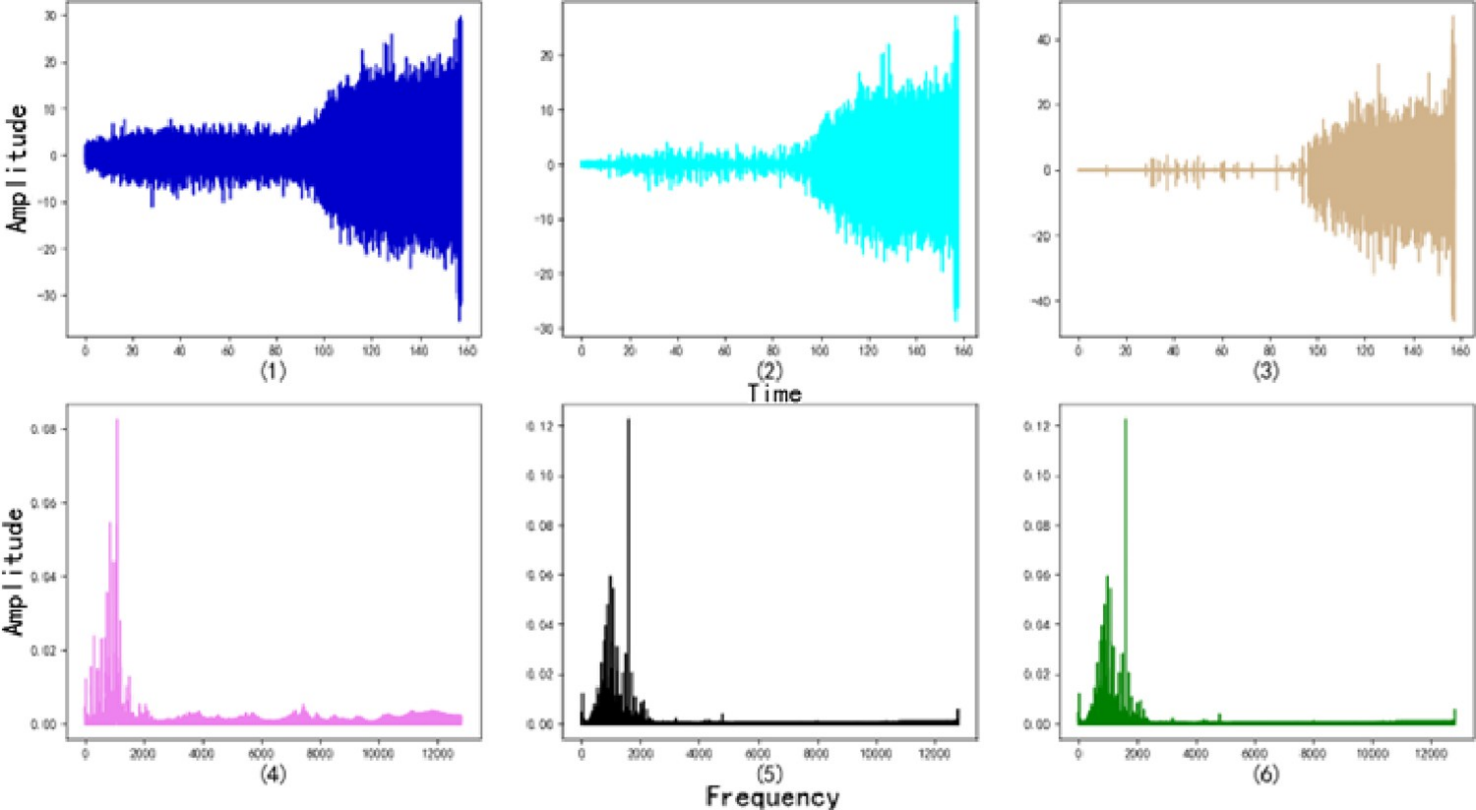
Various WTD methods for denoising effect comparison chart.

The number of wavelet decomposition layers *j* is set equal to 7, and the improved WTD method proposed in this study and the improved WTD (Cited WTD, CWTD) method proposed by Lu Jingyi et al [33] are used to denoise the degradation raw data, as shown in Fig 12.

Fig 12 (1)-(3) respectively show the time domain charts of degradation trend, degradation trend after denoising by CWTD, and degradation trend after denoising by improved WTD. Fig 12 (4)-(6) respectively show the frequency domain charts of degradation trend, degradation trend after denoising by CWTD, and degradation trend after denoising by improved WTD. From Fig 12, we can see that the improved WTD method performs better denoising than the CWTD method. It can significantly suppress the Impact of noise disturbances while retaining information about the bearing degradation status, which can help to accurately judge the beginning moment of the accelerated degradation stage and the failure stage. In other words, it is the ability to accurately separate the fault features from the noise disturbances to achieve a more reasonable FFE effect.

### 4.3 Degradation trend estimation analysis

In this study, the observed variable **X**_*t*_ of the improved MSET method is each wavelet decomposition coefficient obtained by the improved WTD method, which means that $X_{t}=[Ca_{t,7},Cd_{t,7},Cd_{t,6},Cd_{t,5},Cd_{t,4},Cd_{t,3},Cd_{t,2},Cd_{t,1}]$ when *j* = 7. The first 2 seconds of the bearing degradation data are set as the health status, which means the historical moment *m* = 2. The historical memory matrix **D** = [**X**_**1**_,**X**_**2**_]. The remaining degenerate data per 2 seconds is set to the deterioration status, which is the observation vector **X**_*obs*_. The bearing degradation raw data are brought into the proposed DTE method, and a total of 77 degradation status indexes *DR* are obtained.

**Fig 13:**
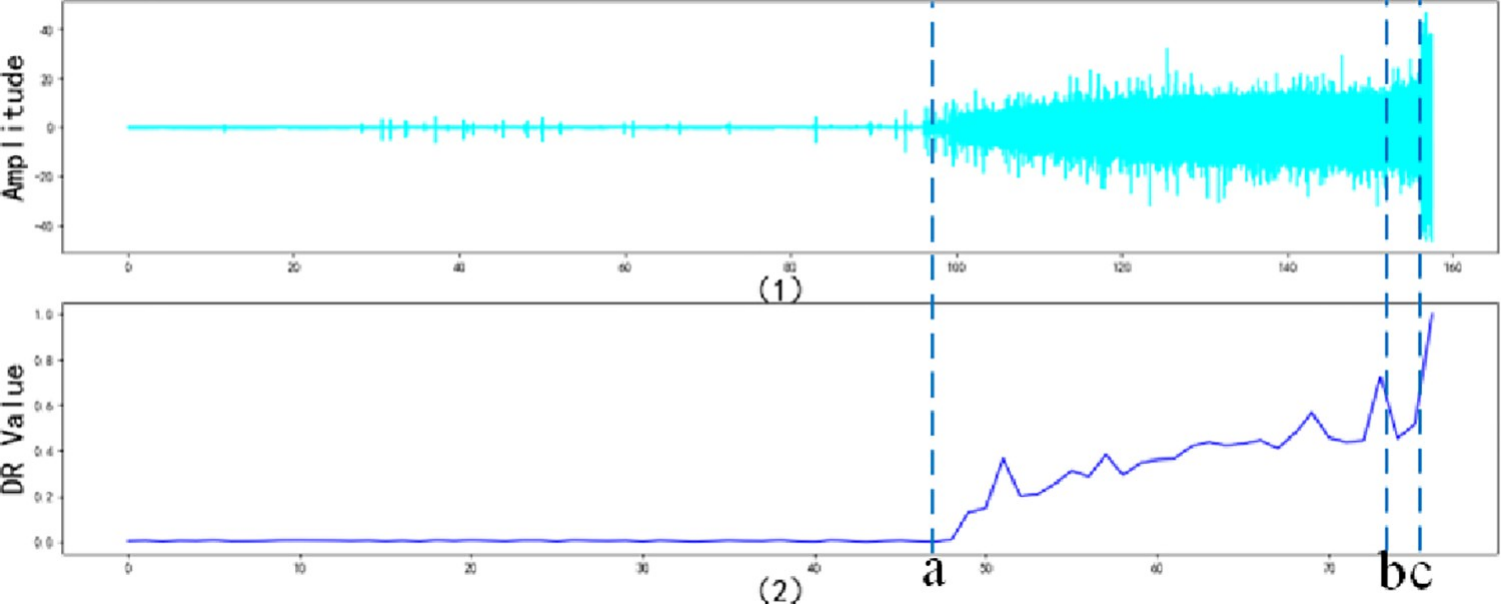
Bearing degradation trend characterization graph.

Fig 13 (1) and (2) show the time domain chart of the bearing degradation trend and degradation trend characterization graph, respectively. We can see that before point *a*, the bearing is in the normal operation stage, and at this stage, where the increase of the degradation status *DR* is less pronounced. Between the points *a* and *c*, the bearing is in an accelerated degradation stage, where the degradation status *DR* increases rapidly. After point *c*, the bearing is in the failure stage, and the degradation status *DR* increases sharply from point *b* to *c*. In other words, the improved MSET method can precisely predict the critical moment of each degradation status and can adjust the degradation status *DR* to warn early at the moment of imminent failure. Meanwhile, Fig 13 also show that the degradation trend of the bearing does not increase monotonically when the bearing is in the accelerated degradation stage, which is between the points *a* and *c*, but there is a slight local fluctuation. This illustrates the generality of the problem presented in this study and the limitations of traditional DTE methods.

For the estimation of system resilience.

Step 1. The expert score for the father nodes and the results are shown in Table 2.

**Table 2:** Father nodes score table.

Father node	level	Father node	level
Materials	High	Maintenance Strategy	High
Load	High	Structure	Medium
Craftsmanship	Medium	Management	Medium
Environment	Low	Assembly quality	High

Step 2. The reliability variable *x*_1_ = 0.850, the recoverability variable *x*_2_ = 0.792, and the robustness variable *x*_3_ = 0.800 are calculated by Eq (16).

**Fig 14:**
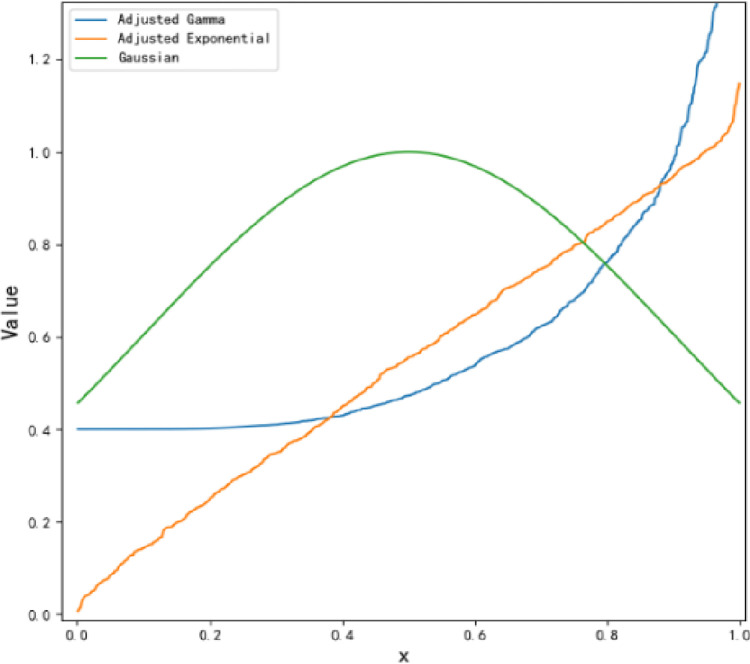
Son nodes membership function diagram.

**Fig 15:**
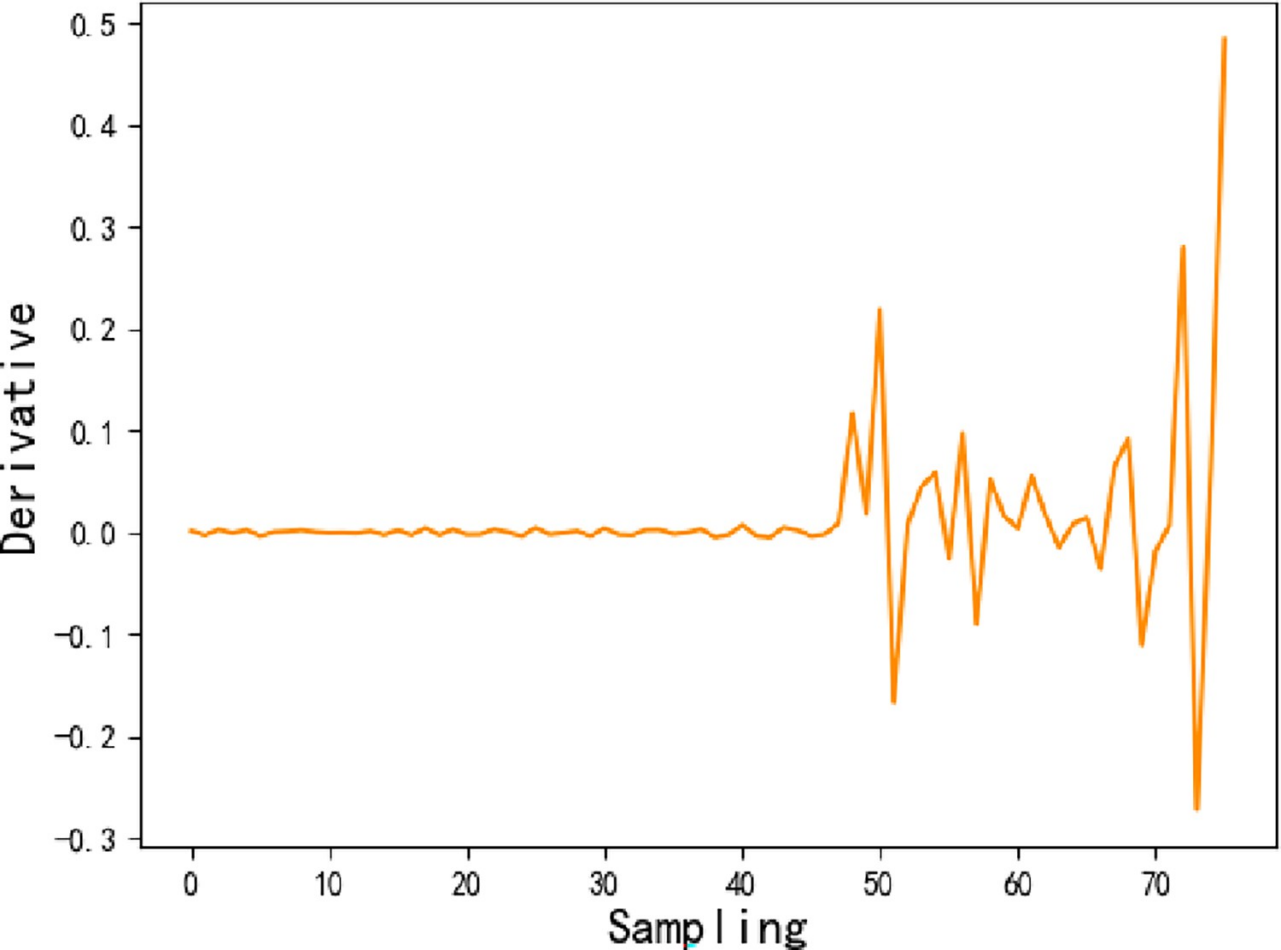
The derivatives of the degenerate function.

Step 3. Let *x*_1_,*x*_2_ and *x*_3_ be the adjusted generalized gamma distribution, adjusted exponential distribution, and normal distribution, respectively, as shown in Fig 14. From Eq (15), its reliability index *P*(*R*_1_|*X*_1_,⋯,*X*_5_) = 0.831, recoverability index *P*(*R*_2_|*X*_2_,⋯,*X*_7_) = 0.855, and robustness index *P*(*R*_3_|*X*_4_,⋯,*X*_8_) = 0.755. At the same time, the derivatives of the degradation trend function *f*, shown in Fig 15, are calculated, as well as the *Sgn*(∂*f*).

Bringing *P*(*R*_1_|*X*_1_,⋯,*X*_5_), *P*(*R*_2_|*X*_2_,⋯,*X*_7_), and *P*(*R*_3_|*X*_4_,⋯,*X*_8_) into Eq (17), then the system resilience *SR*≈0.54, which means that 54% of the performance can be recovered after degradation occurs.

**Fig 16:**
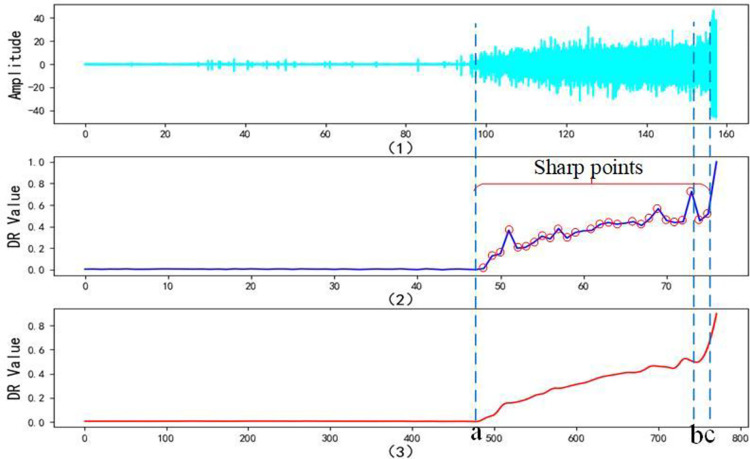
Degradation trend smoothing graph.

Bringing *SR* as an index of system intrinsic capability into Eq (18) corrects the degradation trend. To eliminate the ‘sharp points’ in the *DR* curve where the derivatives are absent, this study uses double CSI to process the data. The number of interpolations is 10 times the amount of original data, implying a total of 770 smoothed degradation status points, as shown in Fig 16. The 38 *DR* values from the 770 are selected for display according to the ratio of 1 in 20, as shown in Table 3.

**Table 3:** Part of the DR value.

Sampling Points	DR	Sampling Points	DR	Sampling Points	DR	Sampling Points	DR
1	0.0036	11	0.0042	21	0.0051	31	0.4196
2	0.0054	12	0.0074	22	0.0049	32	0.4226
3	0.0043	13	0.0082	23	0.0033	33	0.4457
4	0.0054	14	0.0054	24	0.0101	34	0.4732
5	0.0079	15	0.0029	25	0.1453	35	0.4566
6	0.0067	16	0.0049	26	0.2001	36	0.4427
7	0.0068	17	0.0045	27	0.2512	37	0.4552
8	0.0064	18	0.0055	28	0.2842	38	0.9932
9	0.0082	19	0.0084	29	0.2928		
10	0.0084	20	0.0011	30	0.3603		

Fig 16 (1), (2), and (3) respectively show the time domain chart of bearing degradation trend, degradation trend characterization graph, and degradation trend smoothing graph. From Fig 16 and Table 3, we can see that, First, the DTE method proposed in this study can overcome the disadvantages of the traditional methods such as weak generalization ability, lack of rationality in parameter setting, and the requirement of a large amount of data. And it can be adjusted in combination with the system resilience principle, so that the obtained degradation curve is closer to the optimal monotonic curve. Second, the double CSI method can enhance the predictability of the model by eliminating the ‘sharp points’ where the derivative does not exist, without changing the original degradation trend.

By comparing Fig 16 and Table 3 with the degradation trend graph in Ref [47], the proposed DTE method has the following advantages. First, the degradation trend obtained in this study is less fluctuation and better captures the real degradation trend because the system resilience is considered. Second, compared with the traditional DTE method which requires fitting the degradation indexes which reflect the degradation, the method proposed in this paper has a stronger theoretical basis.

## 5. Conclusion

In this paper, we propose a different method of FFE under noise disturbance and DTE with system resilience for rolling bearings. The results of the present paper include the following.

1. This paper demonstrates the effectiveness of the proposed BIC-WTD and DTE method with system resilience correction through the verification of the complexity of the data and the completeness of the amplitude of the noise disturbance distribution.
2. Compared with traditional degradation trend estimation methods, we utilize the system resilience correction method to estimate the degradation trend more accurately, which provides theoretical support for the subsequent application of system resilience to degradation trend estimation.

However, it should be noted that the study in this paper is still at a preliminary level. For the case of noise generated by the superposition of multiple vibration sources, the present method is not applicable.
